# Physical and psychological effects of outdoor fitness equipment training on middle-aged and older adults: study protocol of a randomised controlled trial

**DOI:** 10.1136/bmjsem-2023-001829

**Published:** 2023-12-30

**Authors:** Pablo Jorge Marcos-Pardo, Alejandro Espeso-García, Raquel Vaquero-Cristóbal, Tomás Abelleira-Lamela, Francisco Esparza-Ros, Noelia González-Gálvez

**Affiliations:** 1 CIBIS Research Center, SPORT Research Group (CTS-1024), University of Almeria, Almeria, Andalucía, Spain; 2 Active Aging, Exercise and Health/HEALTHY-AGE Network, Consejo Superior de Deportes, Madrid, Spain; 3 Facultad de Deporte, UCAM Universidad Católica de Murcia, Murcia, Murcia, Spain; 4 Department of Physical Activity and Sport Sciences, Faculty of Sport Sciences, University of Murcia, Murcia, Murcia, Spain; 5 International Chair of Kineanthropometry, UCAM Universidad Católica de Murcia, Murcia, Murcia, Spain

**Keywords:** Physical activity, Aging, Training, Physical fitness, Performance

## Abstract

This project will investigate the effectiveness of an 8-week outdoor fitness equipment (OFE) programme on health parameters in healthy community-dwelling middle and older adults, corresponding to the present paper to the study protocol, which follows a single-blind randomised controlled trial design. The training intervention will involve participation in an OFE programme implemented over 8 weeks, two sessions/week. Rating of perceived exertion, heart rate, training volume and adherence to the exercise programme will be registered each session. The control group will not receive any structured exercise programme. Blinded examiners will evaluate before and after the training programme muscle strength with both maximum knee extension and elbow flexion isometric tests, handgrip strength test and five time sit-to-stand test; cardiovascular fitness with 400-metre walking test; postural stability with the balance tests of the short physical performance battery (SPPB) battery; dynamic balance with Timed Up and Go (TUG) test; body composition with dual-energy X-ray absorptiometry; functional performance by gait speed, TUG and SPPB battery; blood pressure; and sagittal spine disposition and pelvic tilt. Information will also be collected about demographic characteristics, health-related quality of life, satisfaction with life and adherence to the Mediterranean diet questionnaires. After that, sarcopenia, osteoporosis and fracture risk will be calculated. The results derived from this research will increase the knowledge of the effectiveness of OFE training for improving the health of healthy community-dwelling middle and older adults.

**WHAT IS ALREADY KNOWN ON THIS TOPIC**

- Outdoor fitness programmes can have health benefits for middle-aged and older adults in relation to muscle strength, cardiovascular capacity, postural stability and body composition. However, few studies have examined the benefits of outdoor fitness equipment training for older adults.

**WHAT THIS STUDY ADDS**

- This study provides evidence of the effectiveness of an outdoor fitness equipment training programme in improving specific health-related variables in a randomised controlled trial.

**HOW THIS STUDY MIGHT AFFECT RESEARCH, PRACTICE OR POLICY**

- This study proposes a detailed protocol to evaluate the effects of outdoor exercise equipment training in a specific population. The results could be substantial in the development of more effective and accessible interventions and the promotion of active and healthy lifestyles among older adults.

## Introduction

Practicing consistent physical activity is crucial to uphold optimal health and stave off chronic conditions and the effects of ageing in the older population. According to the WHO, physical activity can reduce the likelihood of middle-aged and older people developing chronic diseases such as heart disease, stroke, diabetes, sarcopenia, certain types of cancer by approximately 30–40%, diseases whose incidence increases with increasing age.^1^

Among the different physical activity options for the prevention of the effects of ageing, resistance training has gained popularity in recent years. In this line, some researchers conclude that resistance and balance exercise, with or without nutritional intervention, are the most effective interventions for improving quality of life and physical function in older adults with sarcopenia.^2^

Public areas are ideal for encouraging physical activity and resistance training since they encompass green spaces and outdoor fitness equipment (OFE).^3^ Furthermore, engaging in outdoor physical exercises can improve pulmonary, cardiovascular, cognitive, musculoskeletal and overall systemic health.^4^ Despite this, few studies have examined the benefits of OFE training for older adults.^5^ A recent review demonstrates that OFE training programmes have the potential to improve cardiovascular health, muscle strength, flexibility and balance and reduce falls while improving quality of life, alleviating depression and anxiety and increasing self-esteem.^6^ However, the authors suggest that more rigorous research is needed to understand the psychophysiological and social effects on health comprehensively.

Therefore, OFE training may be a free, feasible and timely way to address various health-related aspects of the ageing process. However, to date, no studies have been found that have analysed the effects of planned OFE training on health in middle and older adults with a randomised controlled trial design. Thus, this project will investigate the effectiveness of an 8-week OFE programme on health parameters in middle and older adults, corresponding the present paper to the design of the study protocol in which it presents an ongoing randomised controlled trial to investigate the effects of a circuit resistance training intervention with OFE on relevant physical and psychosocial performance in healthy community-dwelling middle and older adults.

The hypothesis is that the OFE training for 8 weeks, twice per week, improves physical fitness, body composition, psychological state and prevention of the effects of ageing after the intervention period compared with the daily activities control group (CG).

## Materials and methods

### Study design and setting

This protocol describes an 8-week two-armed parallel single-blind randomised controlled trial with blinded examiners (NCT04958499). This study is designed considering the recommendations of Consolidated Standards of Reporting Trials 7 and Standard Protocol Items: Recommendations for Interventional Trials statements.^8^

**Figure 1:**
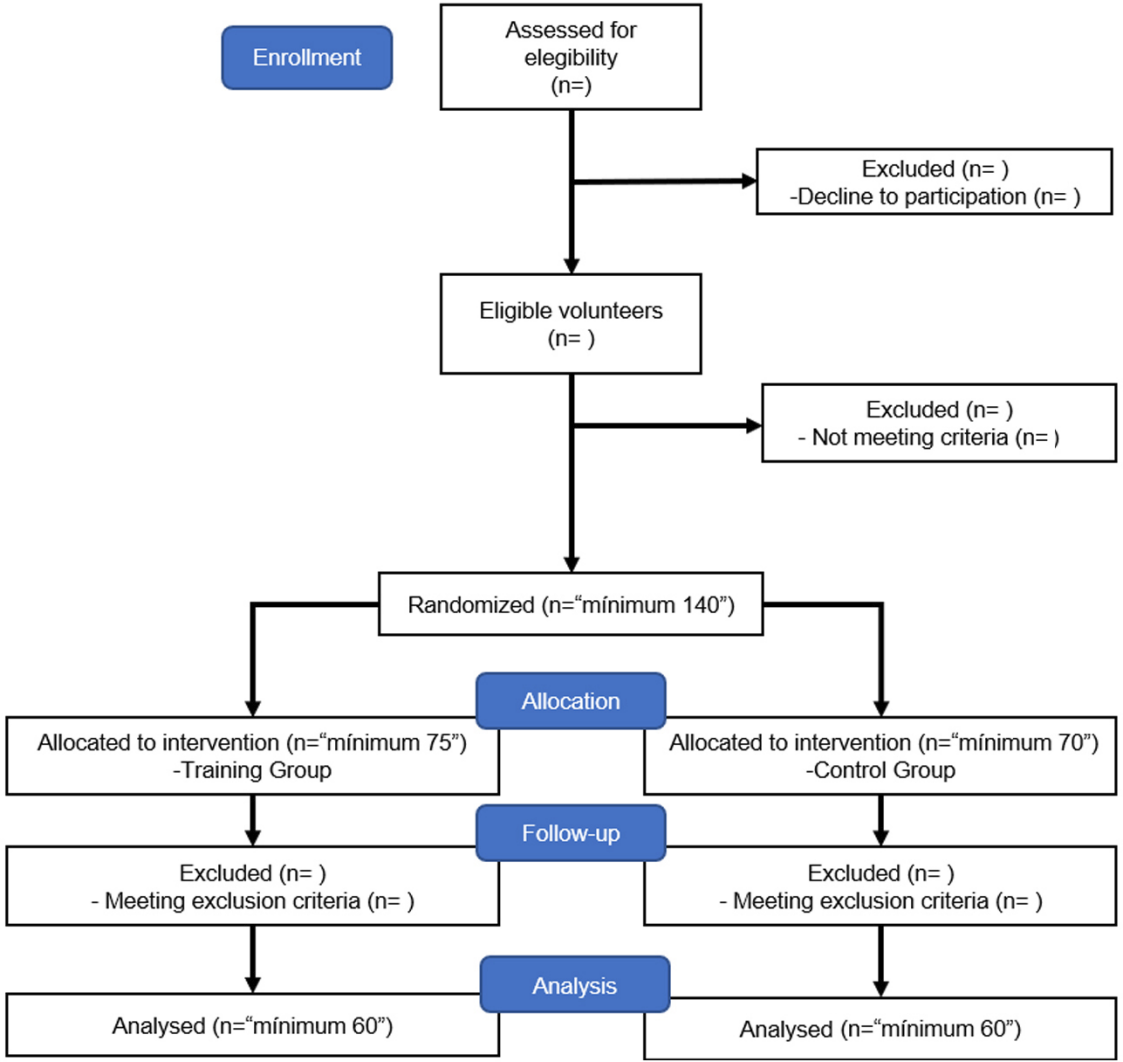
Consolidated Standards of Reporting Trials flow diagram showing the flow of participants through each stage of the study.

Participants will be evenly and randomly divided between the training group (TG) and the CG. The overall study design is summarised in figure 1. The choice of this design is related to the main objective and facilitating the interpretation of the data with intragroup and intergroup.

### Ethics approval and dissemination

All participants will be informed about the study procedures and will give their approval through informed consent before inclusion. All data collected during the study will be confidential. All participants receive a final report with their results to know their values at baseline and the end of the study. The trial will be conducted following the Declaration of Helsinki, and the study has been approved by the institutional ethics committee of the Catholic University of Murcia (CE111908).

Based on previous studies, groups larger than 30 were required for the result to reliably characterise precision error or change during clinical monitoring.^9^ A dropout rate of 16% will be assumed based on previous studies,^10^ so a minimum of 35 participants will be included per group. The RStudio V.3.15.0 software will be used to calculate the sample size. The sample size and statistical power were established considering the SD (4.2) in the maximum voluntary isometric contraction (MVIC).^11^ A significance power of 95% (1-β=0.95) and a significance level of 0.05 will be considered. Ultimately, the sample consisted of a minimum of 60 participants per group. This resulted in an assumed error of 1.06.

### Recruitment

Participants will be recruited through advertisements in health centres, older daycare centres, senior centres and women’s centres in Murcia (Spain). In addition, publications with information about the trial will be made on social networks. All potential participants will be contacted personally by telephone before their inclusion in the study.

### Inclusion and exclusion procedures

To be included in the study, participants will have to meet the following criteria: will be male or female, aged 50 years or older, being physically independent, not having any pathology incompatible with the study, not having participated in a structured exercise programme for at least 1 year and signing the informed consent form. The exclusion criteria will be self-reported substance abuse or dependence disorder (alcohol, non-prescribed medications, etc), being under medical prescription for taking medications that could influence physical performance, suffering from any pathology contraindicated for the exercise programme or requiring specialised medical attention (infectious, renal, pulmonary, coronary diseases, etc), having low adherence during the programme or performing physical exercise outside the study. During the pre-test phase, health personnel will perform an ECG on each participant to corroborate their cardiac health just after signing the informed consent and before the assessment tests.

### Randomisation and allocation

After baseline measurements, participants will be randomly assigned to the TG or CG. A research team member will create a randomisation sequence (1:1) before the trial begins. The investigators who will conduct the assessments are not aware of which group the participants belong to, and participants will be instructed not to disclose the group to which they had been assigned.

### Training group

The TG will perform circuit strength training with OFE in groups of six to eight people. Two sports science graduates will supervise the training programme, which will be carried out for 8 weeks, with two training sessions per week on non-consecutive days (table 1). To increase adherence, a third weekly session will be available for participants who could not attend on their schedule.

**Table 1:** Progression in training during the study

Week	Workouts/week	Sets	Work/rest time (s)	Execution time (concentric-eccentric) (s)	Rest interval (s)
1	2	1	30/45	1–1	120
2	2	2	30/30	1–1	120
3	2	3	30/30	1–1	120
4	2	3	30/30	1–1	120
5	2	3	45/30	1–2	120
6	2	3	45/30	1–2	120
7	2	3	45/30	2–2	120
8	2	3	45/30	2–2	120

.s, seconds.

Over the 8 weeks, both the volume and intensity of the training will be progressively increased. Training load control considers variables such as time under tension, execution time, training density, rest between sets and total volume.

The session structure will be based on timed circuit training. Each session will specify work time, rest time, execution time and sets to perform (table 1). Work and rest times will be monitored through Timebirds Original V.3.12 (Melbourne, Australia). A mobile application metronome (Metronome Beats, UK) programmed at 60 beats per minute will indicate execution time with a series of beeps. Participants will perform repetitions constantly, using the metronome information to control execution time during the concentric and eccentric phases.

**Figure 2:**
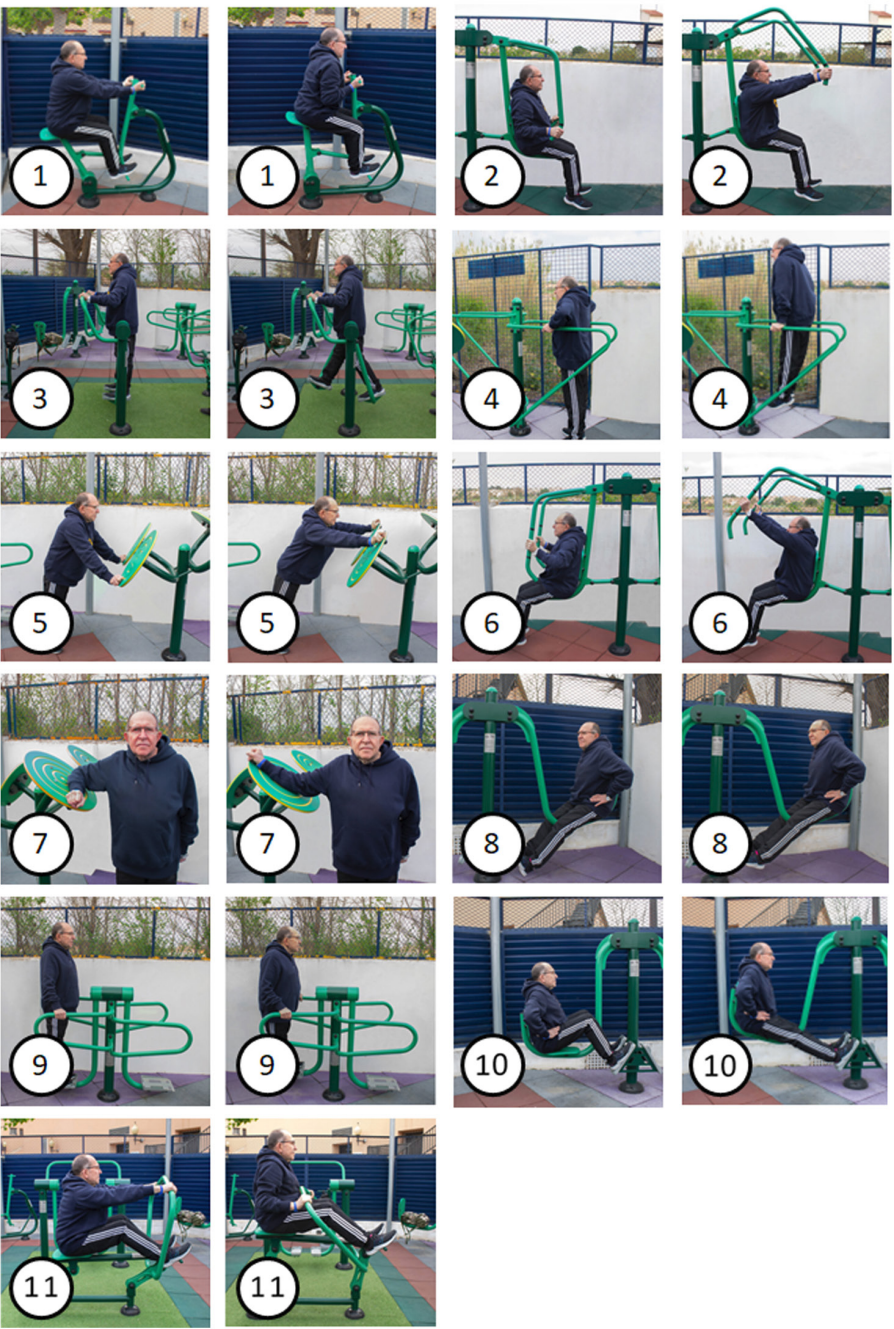
Images of the sequence and execution of the exercises on the different machines. (1) Bonny rider; (2) Gemini Low Grip; (3) Air Walker; (4) Parallel Bars; (5) Flyer Wheels circumduction; (6) Gemini Mid Grip; (7) Flyer Wheels External Rotation; (8) Swing Anke Extension; (9) Surfboard; (10) Swing Leg Extension; (11) Row.

The warm-up will consist of aerobic exercises designed to elevate the heart rate (HR), which include 150 m of walking, increasing the walking speed every 50 m without transitioning to a run, 10 body weight squats and 10 standing push-ups against the wall. Following the aerobic exercises, participants will perform upper and lower joint mobility movements to prepare them for the training. The cool-down phase will involve gentle stretches of the major muscles targeted during the session, with 10 s of stretching per position. The training circuit will comprise 11 exercises conducted on 8 OFE from Entorno Urbano S.L.U (Murcia, Spain), installed at the university facilities. These machines offer different usage modes by varying grips and limb positions. Accordingly, the training circuit will be composed of the following machines in sequence: Bonny rider (the handle will be pulled using both hands with a supine grip, with the elbow being flexed and the shoulder being extended), Gemini Low Grip (the handle will be pushed with grips held at a low height, with the elbow being extended and the shoulder being flexed), Air Walker (hip flexion-extensions will be performed on both platforms with the torso being kept upright. A brief isometric hold will be done at the end of the motion), Parallel Bars (the body’s weight will be lifted by the elbow being extended from a flexed position. As an adaptation, triceps push-ups will be done sideways to the machine with a prone grip), Flyer Wheels circumductions (circumductions will be performed on the Flyers Wheels apparatus with elbows kept straight and grips being held, using shoulder mobility), Gemini Mid Grip (the handle will be pushed on the Gemini equipment with grips held at mid-level, by the elbow being extended and the shoulder being flexed), Flyer Wheels external rotation (internal and external rotations will be executed on the side of the Flyers Wheels machine, with the elbow being supported and the grip being held), Swing ankle extension (with the knee kept straight, the metatarsal will be rested on the platform’s lower edge and the ankle will be alternately flexed and extended), Surfboard (a pendulum motion from the waist will be done in the frontal plane while the handle is being held on the platform. An isometric hold will be performed at the motion’s furthest point), Swing leg press (resistance will be pushed against on the Swing apparatus with the knees being extended, while seated with feet on the platforms) and Row (on the seat, the movement will be performed by bending the elbow and shoulder, keeping the torso upright) (figure 2).

**Figure 3:**
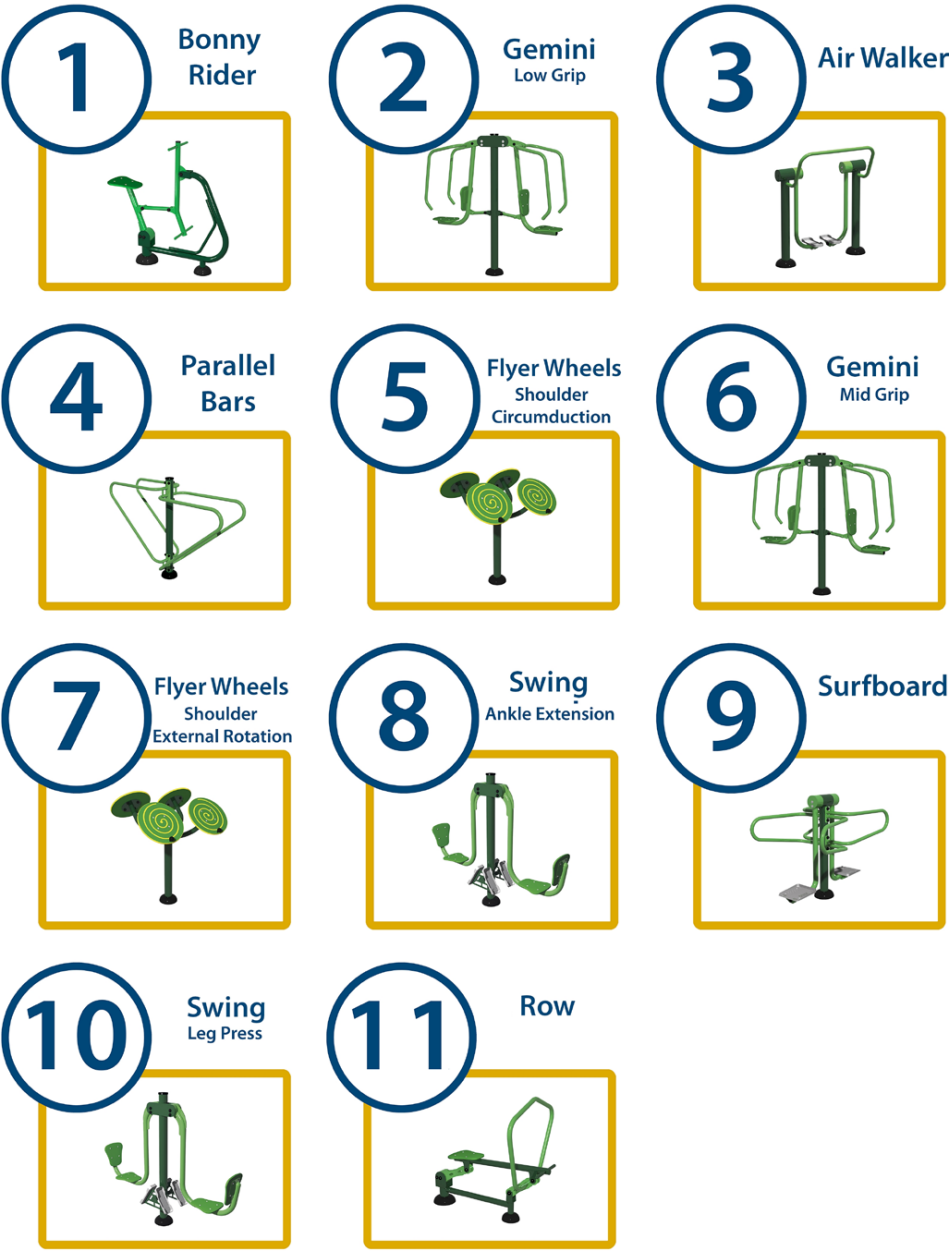
Images of flashcards indicating the sequence of use for the outdoor fitness equipment during the training programme.

Each machine will be numbered with a sign to indicate the execution order to the participants (figure 3), allowing for alternating work on different muscle groups to prevent excessive localised fatigue. The starting exercise for each participant and session will be randomised to avoid potential influences on ratings of perceived exertion (RPE) and HR. The initial machine will be recorded for each participant in each session.

At the session’s end, data on HR will be collected using a wrist-worn HR monitor, the Polar M430 (Polar Electro, Kempele, Finland), and participants’ RPE after each exercise and at the end of the session will be recorded. Potential exercise adaptations will be based on participants’ functional and physical capacity, assessed by an expert committee and considering the machines’ everyday use.^12^

### Control group

Individuals assigned to the CG (figure 1) will be instructed to continue their daily activities throughout the study. Participants’ habits and physical activity levels will be monitored over the 8-week study period using specific inquiries incorporated within the socio-demographic questionnaire.

### Data collection and measures

Measurements will be performed in the laboratory of the research group available at the university. Participants will be asked not to stretch or warm up before the tests. Measurers will be maintained from pre-test to post-test to avoid the influence of inter-rater error on the measurements made. Experts in the performance of the specified tests will evaluate all measurements. The tests will be performed in a random order. Variables collected and the assessment schedule during the study are shown in table 2.

**Table 2:** Variables were collected, and assessments were scheduled during the study

	Training group			Control group		
	Baseline	Intervention	Post	Baseline	Intervention	Post
Consent	X			X		
Demographic survey: Age, sex, occupation, weekly physical activity, pathologies and medication	X			X		
Health-related quality of life: 36-Item Short Form Survey	X		X	X		X
Satisfaction with life: Satisfaction with Life Scale	X		X	X		X
Adherence to the Mediterranean diet: Prevention of Mediterranean Diet Score	X		X	X		X
Body composition: Height and weight Full body composition (DEXA) Bone mineral density (DEXA)	X		X	X		X
Blood pressure	X		X	X		X
Heart electrical activity: ECG	X			X		
Muscular strength: Isometric handgrip strength test Isometric knee extension strength test Isometric elbow flexion strength test	X		X	X		X
Cardiovascular fitness: 400 metres walking test	X		X	X		X
Postural stability: Centre of pressure (COP) in side-by-side test Centre of pressure (COP) in semi tandem test Centre of pressure (COP) in tandem test	X		X	X		X
Functional capacity: Short physical performance battery (SPPB) Time Up and Go Test	X		X	X		X
Risk of falls: Gait speed test	X		X	X		X
Risk of sarcopenia: European Working Group on Sarcopenia in Older People Consensus	X		X	X		X
Sagittal spine disposition and pelvic tilt: Spinal mouse relaxed standing position test Spinal mouse relaxed sitting position test	X		X	X		X
Rating of perceived exertion: 1–10 Borg rating of perceived exertion		X				
Effort and heart rate		X				
Training volume		X				
Adherence		X				

DEXA, dual-energy X-ray absorptiometry.

### Primary outcomes

#### Maximum muscular strength

Both maximum knee extension and elbow flexion strengths will be evaluated under the same isometric conditions. For the knee extension test, participants will be seated in a chair specifically designed for the measurement of isometric knee extension strength (ES-1295254) equipped with a force transducer (Musclelab, Ergotest, Norway), maintaining both a hip and knee angle at 90 degrees. Participants will be directed to exert maximum strength against a pad above their ankle joint. For the elbow flexion test, participants will stand upright, holding a bar with both hands in a supine position, with the elbow at a 90-degree angle. Participants will be directed to exert maximal strength against the bar.

Following a previous protocol, participants will sustain exertion for 2–3 s during both assessments.^13^ The software (Musclelab, Ergotest, Norway) will quantify the maximum voluntary isometric contraction and the rate of force development. Each participant will complete three trials for each assessment, and the best one will be used.

#### Handgrip strength test

Handgrip strength (HG) test will be measured standing with the arms at the sides. The participants will perform one repetition in each hand to familiarise themselves with the device and the test. Each participant will be asked to squeeze the grip with maximal strength with both hands for 3 s. The highest peak strength (kg) recorded between the three attempts will be considered for analysis. A digital grip strength dynamometer will be used for this (TKK 5401; Takei Scientific Instruments, Tokyo, Japan). This measure has been associated with frailty in older people.^13 14^

#### Cardiorespiratory fitness

The 400 m walking examination is deemed a suitable and reliable test for approximating cardiorespiratory fitness (CRF).^14^ So CRF will be evaluated by measuring the time needed to complete 10 laps as rapidly as feasible on a 20 m long track. Participants will be directed to walk at their maximum pace, refraining from running.^14^ The maximum and average HR during the test will be evaluated, and the HR with the test will be finished. Also, the HR 2 min after the test will be recorded as the CRF recovery variable. A single attempt at the test will be done.

#### Postural stability

A force platform will assess postural stability during quiet standing (Musclelab, Ergotest, Norway). The short physical performance battery (SPPB) balance protocol will be followed.^15^ Participants will be maintaining a stationary stance for 30 s with arms at their sides, repeated three times in each foot position, following this order: (1) feet together with eyes open; (2) semi-tandem stance (one foot’s ankle behind the joint of the other foot) with eyes open; and (3) tandem stance (one foot’s ankle directly behind and touching the other foot’s ankle) with eyes open. Measurements such as the total centre of pressure displacement, anteroposterior and mediolateral amplitudes of the centre of pressure and the area of the centre of pressure displacement will be obtained from analyses conducted with Musclelab software (Musclelab, Ergotest, Norway).

#### Dynamic balance

The Timed Up and Go (TUG) test will assess the dynamic balance. During the TUG, participants will stand up from a chair, walk 3 m, turn around and sit back down in the chair while timed (38). Two attempts will be made, and the final value will be the best.

#### Body composition

An accredited anthropometrist will measure kinanthropometric parameters related to height and body mass according to the guidelines of the International Society for the Advancement of Kinanthropometry.^16^ The portable Leicester HR 001 stadiometer (Tanita, Illinois, USA) will be used to measure the height (cm) with a precision of 1 mL, while body mass (kg) will be assessed in light clothing and barefoot using a Tanita BC-545N electronic scale (Tanita, Illinois, USA) with a precision of 0.1 kg. Each measurement will be taken twice. If both measurements have a difference of less than 1%, their average will be calculated. Conversely, if the difference exceeds 1%, a third measurement will be taken, and the median will be considered.

Body composition will be evaluated using dual-energy X-ray absorptiometry (DEXA) through a Hologic QDR 4500A densitometer (Hologic, Massachusetts, USA). A whole-body scan will be conducted to assess lean and fat mass percentages.

Additionally, a second scan of the right proximal femur (femoral neck, trochanter region and ward area) will be performed to evaluate bone mineral density levels, osteoporosis risk and WHO-FRAX. The same certified technician will conduct all measurements and densitometry analyses following key guidelines.^17^ Participants will be evaluated in a supine position with hips in internal rotation and feet in adduction to enhance femoral neck visibility. All participants will be instructed to use the restroom before the test and wear light clothing without metallic objects or shoes.^17^ A single attempt at the test will be done.

#### Functional performance

Functional performance will be measured by 10 m of gait speed, the TUG and the SPPB.^18^

Gait speed will be assessed using a 10-metre test. Participants will be asked to walk at maximum speed without running, and the total time taken to cover the 10 m distance will be measured with two photocells (Witty, Microgate, Bolzano, Italy). The test will be performed twice, and the best one will be used.

The SPPB encompasses the postural stability assessment, five-time chair stand test and 4 m gait walk.^18^ The five times sit-to-stand test will evaluate the gauges and the duration the subject takes to transition five times from a seated posture to a standing posture with utmost speed, refraining from using their arms. The test will be performed twice, and the best one will be used. In the 4 m gait walk, participants will be asked to walk at maximum speed without running and the total time taken to cover the 4 m distance.^18^ To ensure a dependable measurement, it will be measured by two photocells (Witty, Microgate, Bolzano, Italy). The test was performed twice, and the best one was used.

Of the tests included in the SPPB, each test allowed for a potential accumulation of up to four points, yielding a score spectrum of 0– 12 points.^15^

#### Sarcopenia

The HG and chair stand tests will assess muscle strength (muscle quality). Muscle quantity will be measured through appendicular skeletal muscle mass with DEXA values, and functional performance will be measured by 4 m gait speed, TUG and the SPPB battery. The risk associated with each variable will be defined in connection with the EWGSOP2 (European Working Group on Sarcopenia in Older People) sarcopenia cut-off point.^18 19^

### Secondary outcomes

#### Demographic survey

Participants will be asked about their basic socio-demographic data at the baseline. They will also be asked about their daily physical activity level, number of falls and weight loss in the last year and information regarding their health status, such as medication and pathologies.

#### Health-related quality of life

The 36-item Short-Form Health Survey (SF-36) will be employed to assess health-related quality of life. The SF-36 evaluates physical and mental health domains across eight concepts: physical functioning, physical role functioning, bodily pain, general health perceptions, vitality, social role functioning, mental health and emotional role functioning. The validated Spanish version^20^ will be used in this study. A higher score indicates a better quality of life.^20^

#### Satisfaction with life

The Satisfaction with Life Scale questionnaire will be used to assess life satisfaction, specifically in its Spanish version.^21^ This instrument consists of five items responded to using a 5-point Likert scale. These items are related to an individual’s perception of achievements relative to their expectations. A higher value indicates greater life satisfaction.^21^

#### Adherence to the Mediterranean diet

Adherence to the Mediterranean diet will be assessed using the score obtained from the validated 14-item questionnaire of the Prevention of Mediterranean Diet (PREDIMED) study.^22^ A higher score indicates greater adherence to the Mediterranean diet, with a score of ≥9 considered high adherence.^22^

#### Blood pressure

Blood pressure will be measured using an automatic blood pressure monitor (OMRON model HEM-7113, Osaka, Japan), following the general guidelines.^23^ The participant will sit comfortably and quietly for 5 min before the first measurement, with the second measurement taken 2 min later. The final values of systolic blood pressure, diastolic blood pressure and pulse rate were the average of the two measurements. Subjects will be informed to abstain from vigorous exercise for 24 hours, avoid caffeine intake for 4 hours and refrain from tobacco use 30 min before the measurements.

#### Leg muscle strength

The chair stand test will be employed to quantify the leg muscle strength. This test will be carried out following the protocol of previous investigations.^18^

#### Risk of falls

Gait speed is a powerful predictor of fall risk.^24^ Gait speed will be assessed using a 10-metre test.

#### Sagittal spine disposition and pelvic tilt

The sagittal spine disposition and pelvic tilt will be measured using the Spinal Mouse system (Idiag, Fehraltdorf, Switzerland). This device allows the angular measurement of the different spinal curvatures in a non-invasive way. An assessment of the sagittal spine disposition in relaxed standing and sitting will be performed, following the protocol from previous studies.^25^ A positive value implies kyphosis for thoracic and lumbar curvatures and an anteversion for pelvic tilt. In contrast, a negative value implies lordosis for thoracic and lumbar curvatures and a retroversion for pelvic tilt. Each position will be evaluated three times, the final value being the mean of both values.^25^

#### Rating of perceived exertion

The Borg CR-10 RPE scale will be used to measure the perception of exertion among participants.^26^ Participants will be instructed to evaluate their perceived exertion for the entire session and their general perception of exertion for each machine immediately after the session conclusion.

#### Effort and heart rate

The training intensity will be registered by monitoring the HR throughout the sessions. For this purpose, Polar M430 (Polar Electro, Kempele, Finland) wristband HR monitors will be employed. After each session, the average, peak and final HRs will be collected.

#### Training volume

The volume will be systematically registered for each training session. This includes the duration in seconds of the workout, the associated rest intervals and the number of repetitions and sets performed by each participant.

#### Adherence to the exercise programme

In the TG, each participant’s adherence to the exercise programme will be monitored. Adherence will be measured as the percentage of exercise sessions performed and/or the total number of scheduled sessions.

## Patient and public involvement

Patients and/or the public will be involved in this research’s design, performance, reporting or dissemination plans.

## Statistical analysis

The qualitative variables will be summarised in frequency. In contrast, the quantitative variables will be summarised using mean and SD for the TG and CG across the assessment time points. To ensure rigorous statistical evaluation, special attention has been given to controlling the type I (alpha) error and Bonferroni’s correction will be used to achieve p=0.005 for statistical significance. To compare characteristics between and within groups, the principle of intention to treat will be applied, with adjustments made to adhere to the exercise programme. The following tests will be employed to assess baseline characteristic differences between the two groups: Student’s t-test for parametric numerical variables, Mann-Whitney test for non-parametric variables and χ^2^ test for categorical binary variables.

To compare changes within and between the TG and CG across baseline and follow-up assessments, the subsequent tests will be used: for variables exhibiting normal distribution, repeated measures analyses of variance. Given the multiple comparisons involved, post hoc analyses will be conducted using t-tests with Sidak corrections to control the type I error rate; for non-parametric variables, the Friedman test will be followed by the Wilcoxon test, and, to address the multiple comparisons, the Bonferroni correction will be applied.

SPSS V.25.0 statistical software will be used in all the analyses. A p value<0.05 will be used to determine significant differences; however, this will be adjusted appropriately in the presence of multiple comparisons, as detailed above.

## Conclusions

This protocol presents a randomised controlled trial to evaluate the efficacy and safety of an exercise programme with OFE designed to improve physical and psychological health variables.

Exercise and strength training are essential to prevent or reverse disease, maintain or improve health and prevent loss of function in adults, middle-aged and older people. This is where free and free training with OFE in outdoor spaces can be relevant to health. However, no specific indications are available on the appropriate training programme and which machinery can be safe and effective for their physical, psychological and social health. A design study is presented in this research, the results of which are as follows, which could provide evidence to physicians, exercise trainers and policymakers on the best strategy to ensure safe and effective outdoor exercise, establishing parameters for prescribing and monitoring a training programme with OFE, while respecting individual progress.

### Patents

The results of this study were taken into account for the design of new machines that will more effectively and safely, with the following registered patent: Publication n° ES1296848 (U); (http://invenes.oepm.es/InvenesWeb/detalle?referencia=U202231979) and ES1296869 (U); (http://invenes.oepm.es/InvenesWeb/detalle?referencia=U202231980) (accessed on 2 August 2023) and others that are currently under patent review.

## Data Availability

No data are available.
